# Cyclic adenosine monophosphate response element-binding protein transcriptionally regulates CHCHD2 associated with the molecular pathogenesis of hepatocellular carcinoma

**DOI:** 10.3892/mmr.2015.3256

**Published:** 2015-01-26

**Authors:** RUI SONG, BIAO YANG, XUESONG GAO, JINQIAN ZHANG, LEI SUN, PENG WANG, YIXING MENG, QI WANG, SHUNAI LIU, JUN CHENG

**Affiliations:** 1 Institute of Infectious Diseases, Capital Medical University, Beijing 100015, P.R. China; 2 Beijing Key Laboratory of Emerging Infectious Diseases, Beijing Ditan Hospital, Capital Medical University, Beijing 100015, P.R. China; 3 Key Lab of Environmental Pollution and Microecology, Shenyang Medical College, Shenyang, Liaoning 110034, P.R. China; 4 Department of Pathology, Beijing Ditan Hospital, Capital Medical University, Beijing 100015, P.R. China

**Keywords:** CHCHD2, hepatocellular carcinoma, cyclic adenosine monophosphate response element-binding protein, hepatitis C virus, nonstructural protein 2, promoter

## Abstract

The function of the novel cell migration-promoting factor, coiled-coil-helix-coiled-coil-helix domain containing 2 (CHCHD2) in liver cancer remains to be elucidated. The aim of the present study was to elucidate the role of CHCHD2 in liver carcinogenesis. Immunohistochemistry was performed on patients with hepatocellular carcinoma (HCC) and suppression subtractive hybridization (SSH) was used for screening differentially expressed genes in the HepG2 cell cDNA library. Chronic hepatitis C virus (HCV) infection frequently leads to liver cancer. The HCV NS2 protein is a hydrophobic transmembrane protein that is associated with certain cellular proteins. Detailed characterization of the nonstructural protein 2 (NS2) of the HCV was performed with respect to its role in transregulatory activity in the HepG2 cell lines. A gel electrophoresis mobility shift assay and a chromatin immunoprecipitation assay were used to confirm the presence of cyclic adenosine monophosphate response element-binding protein (CREB), a transcriptional factor, which specifically interacts with the CHCHD2 promoter. CHCHD2 was highly expressed in the HCC specimens and was consistent with tumor markers of HCC. CHCHD2 was identified by SSH in the HepG2 cells. NS2 upregulated the expression of CHCHD2 by monitoring its promoter activities. The promoter of CHCHD2 contained 350 bp between nucleotides −257 and +93 and was positively regulated by CREB. In conclusion, the results of the present study indicated that CHCHD2 may be a novel biomarker for HCC and that CREB is important in the transcriptional activation of CHCHD2 by HCV NS2.

## Introduction

The hepatitis C virus (HCV) is a member of the Flaviviridae family of positive-strand RNA viruses and encodes 10 proteins, including three structural proteins (the core protein and the envelope glycoproteins E1 and E2) and seven nonstructural (NS) proteins (NS2, NS3, NS4A, NS4B, NS5A and NS5B) (1–3). The NS2 protein, derived from the cleavage of NS2/3, is inserted into the endoplasmic reticulum membrane through its N-terminal hydrophobic domain (4). NS2 is a hydrophobic protein containing several transmembrane segments in the N-terminal region. NS2 may also be involved in modulating cellular gene expression in infected cells (5,6), although the molecular mechanisms remain to be elucidated.

Suppression subtractive hybridization (SSH) has been previously used to successfully identify and isolate differentially expressed genes (7), particularly the isolation of rare transcripts. To assign a role for NS2, the present study aimed to identify cellular proteins, which interacted with NS2. Coiled-coil-heli x-coiled-coil-helix domain-containing protein 2 (CHCHD2) is a member of a protein family containing a (coiled-coil 1)-(heli x 1)-(coiled-coil 2)-(helix 2) (CHCH) domain (8,9). Our group revealed that the protein was expressed in HCV (10); however, to the best of our knowledge, its expression in liver cancer had not been identified. The present study therefore aimed to investigate CHCHD2 expression in liver cancer. The first and second coiled-coil regions of the CHCH domain have a fixed length of 10 amino acid (aa) and a variable length of 5–10 aa, respectively. The second coiled-coil region may act as a bridge when the two helices fold towards each other. Each α-helix within the CHCH domain contains two cysteine aas (11), which are separated either by nine residues (CX9C motif) (11) observed in the mitochondrial intermembrane space protein Mia40, cytochrome c oxidase copper chaperone, cyclooxygenase 1 and cytochrome c oxidase subunit VIIa or by three residues (CX3C motif) observed in the translocase of inner membrane proteins. Two interhelica l disulfide bonds may contribute to the formation and stabilization the protein tertiary structure (9).

The transcription factor cyclic adenosine monophosphate (cAMP) response element-binding protein (CREB) is a member of a leucine zipper class of transcription factors, which specifically recognizes the cAMP-response element (CRE) promoter site (12). CREB is a target of other signaling pathways and is activated by a diverse array of stimuli, including peptide hormones, growth factors and neuronal activity, which activate a variety of protein kinases, including protein kinase A (PKA), PKC, mitogen-activated protein kinases (MAPKs) and Ca^2+^/calmodulin-dependent protein kinases (13,14). Previous studies have indicated that the phosphorylation of Ser133 is required for CREB-induced gene transcription. Phosphorylation at Ser133 may induce the translocation of cytoplasmic CREB to the nucleus. Phosphorylated CREB within the nucleus may lead to transcriptional activation by promoting interaction with components of the basal transcription machinery, including transcription factor II D and RNA polymerase II (15,16). In addition, there are residues in addition to Ser-133 and the post-translational modifications of CREB (17). CREB regulates several cellular functions, including inflammation, cell proliferation, differentiation, adaptation and survival. However, the role of CREB in regulating the expression of CHCHD2 remains to be elucidated.

The function of CHCHD2 is complex and it is likely that the control of CHCHD2 occurs at multiple levels. However, the mechanisms regulating the expression of CHCHD2 remain to be elucidated.

## Materials and methods

### Clinical specimens

Patients with a diagnosis of HCC underwent hepatectomy surgery between January 2009 and May 2012. In total, 110 samples were obtained from these surgeries. The mean patient age was 50.9±9.2 years, 87 patients were male and 23 were female. All were obtained from paraffin blocks from Beijing Ditan Hospital. HCC paraffin blocks and frozen tissues were obtained from the archives of the Department of Pathology, Beijing Ditan Hospital, Capital Medical University (Beijing, China) with approval from the Institutional Review Board. A total of 110 HCC tumor and 50 noncancerous liver tissue samples, which included 20 patients with cirrhosis, 20 patients with other liver diseases, including chronic hepatitis, drug-induced liver injury and nonalcoholic steatohepatitis and 10 healthy controls were obtained.

Clinical follow-up data, including serum levels of α-fetoprotein (AFP) and other blood biochemical indices were obtained from the patients’ records and retrospectively from case review.

The use of human specimens in the present study was approved by the Ethics Committee of Beijing Ditan Hospital according to the Declaration of Helsinki. All necessary consent was obtained from any patients involved in the present study, including consent for involvement in the study and consent to publish.

### Immunohistochemistry

The tissue specimens were routinely processed and stained with hematoxylin and eosin (H&E). The diagnosis of HCC was based on pathology according to the International Working Party criteria (18) and tumor grading was assessed on the H&E-stained sections according to Scheuer’s system (18).

The HCC biopsy specimens were subjected to routine immunohistochemical staining using a monoclonal antibody according to a previously described method (19). Briefly, sections were deparaffinized in xylene and rehydrated in decreasing concentrations of ethanol (100, 90, 80 and 70%). Slides were incubated in 3% H_2_O_2_, 50% methanol in wash buffer [phosphate-buffered saline (PBS) and 0.1% Tween-20] to quench endogenous peroxidases. Sections were subsequently incubated with primary antibodies for 1h at room temperature. Primary antibodies were purchased from Abcam (Cambridge, UK), and included monoclonal mouse anti-human CD34 (1:50), GPC3 (1:10), GS (1:100), HSP70 (1:100), Ki-67 (1:75) and CHCHD2 (1:300), and the PEG10 (1:1,000) anti-mouse secondary antibody. All antibodies were obtained from Dako North America, Inc. (Carpinteria, CA, USA).

Immunoreactivity, which was defined as the number of positive tumor cells observed in the total number of tumor cells, was scored independently by two researchers. The numbers of CHCHD2-positive and -negative HCC cells were counted under a light microscope (80i; Olympus Corp., Tokyo, Japan) at a magnification of ×400, with only the cells exhibiting brown nucleoli considered CHCHD2-positive. For each slide, between 7 and 10 microscopic fields were randomly selected. The positive scores were the categorized into weak staining with staining in only one nucleolus; moderate staining, with staining in more than one nucleolus; and strong staining, with staining present in the nucleus and nucleolus of the tumor cell. The average percentage of CHCHD2-positive HCC cells was then calculated for each group.

### Cell culture

The HepG2 cells (China Infrastructure of Cell Line Resource, Beijing, China) were cultured in Dulbecco’s modified Eagle’s medium (DMEM) supplemented with 10% fetal bovine serum (Shanghai BioAsia Biotechnology, Shanghai, China), 100 U/ml penicillin G and 100 *μ*g/ml streptomycin (North China Pharmaceutical Co. Ltd, Shijiazhuang, China) in a humidified chamber at 37°C and 5% CO_2_. The cells were seeded into 48-well plates, grown to 90% confluence and transiently transfected using Lipofectamine 2000 (Invitrogen Life Technologies, Carlsbad, CA, USA) according to the manufacturer’s instructions.

### Screening of SSH, clones and identification of the CHCHD2 gene

A subtracted cDNA library was constructed by SSH using a PCR-select™ cDNA subtraction kit (Clontech Laboratories, Inc., Mountain View, CA, USA) according to the manufacturer’s instructions. A total of 2 *μ*g mRNA was used for each first-strand cDNA synthesis. The cDNA obtained from the HepG2 cells transfected with pcDNA3.1 (−)−NS2 (genotype 1b; Beijing Key Laboratory of Emerging Infectious Diseases, Beijing Ditan Hospital) was used as a tester and the cDNA from the HepG2 cells transfected with pcDNA3.1 (−) was used as a driver. Subsequently, the subtracted PCR products were cloned into pGEM-T Easy vectors (Promega Corp., Madison, WI, USA). The ligation reactions were then transformed into chemically competent DH5α cells (China Infrastructure of Cell Line Resource) using standard molecular biology techniques.

Following sequencing of the positive colonies (Shanghai BioAsia Biotechnology, Shanghai, China), the basic local alignment search tool (BLAST) server (http://blast.ncbi.nlm.nih.gov/Blast.cgi) at the National Center for Biotechnology Information (Bethesda, MD, USA) was used to identify nucleic acid homology.

### Expression of CHCHD2

The HepG2 cells were lysed for 36 h by transient transfection with Mammalian Cell Lysis reagent (Thermo Fisher Scientific, Waltham, MA, USA) containing 1% phenylmethyl sulfonylfluoride and centrifuged for 5 min at 12,000 × g. The lysates were then diluted in the supernatant using sodium dodecyl sulfate (SDS) buffer and separated using SDS-PAGE (Thermo Fisher Scientific). Following transfer onto a nitrocellulose membrane (Millipore, Billerica, MA, USA), the blots were inhibited for 2 h with 5% milk-PBST (PBS+0.05% Tween). For the detection of CHCHD2, rabbit CHCHD2 antibody (1.0 mg/ml) (20) was diluted (1:1,000) in PBST buffer. The mouse anti-β-actin antibody (Santa Cruz Biotechnology, Inc., Austin, TX, USA) was diluted (1:2,000) in PBST buffer. After 10 h incubation at 4°C and extensive washing with PBST buffer, horseradish peroxidase-conjugated goat-anti-rabbit immunoglobulin (Ig) secondary antibody (Bio-Rad, Hercules, CA, USA) was added (1:1,000) in 5% milk-PBST buffer for 2 h at room temperature, which was also used to detect the protein expression of β-actin. Following additional washing, the blots were developed using an enhanced chemiluminescence substrate system (Pierce ECL Western Blotting Substrate; Thermo Fisher Scientific).

### RNA extraction and reverse transcription quantitative polymerase chain reaction (RT-qPCR) analysis

The total RNA was extracted from the cultured cells using TRIzol reagent (Promega Corp.) according to the manufacturer’s instructions. A total of 0.1 *μg* RNA from each sample was used to generate cDNA by reverse transcription using the One-step RT-PCR kit (Takara Bio, Inc., Shiga, Japan). A Taqman RT-qPCR assay was performed on an ABI Prism 7500 system (Applied Biosystems Life Technologies, Foster City, CA, USA) according to the manufacturer’s instructions. β-actin was used as a reference for normalizing the data. The cycling conditions were as follows: 95°C for 30 sec, 40 cycles of 95°C for 5 sec, 60°C for 34 sec and finally 60°C for 1 min. The CHCHD2 primers and probe used were as follows: Forward, 5′-GCATCATCCCCATTCCGAAGG-3′ and reverse, 5′-ACCTGATTGGCTCGCTCTCC-3′; probe: 5′-CTCCGGCTGCACCTCGCTTGGC-3′. The GAPDH primers used were: Forward, 5′-ACAGCCTCAAGATCA TCAGCA-3′ and reverse, 5′-ATGAGTCCTTCCACGATA CCA-3′; probe: 5′-GTGCTAAGCAGTTGGTGGTGCAGG A-3′. Primers were obtained from Promega Corp.

### Molecular cloning of the CHCHD2 promoter

The genomic DNA was isolated from the HepG2 cells using a Genomic DNA Purification kit (Promega Corp.). A series of 5′-fanking DNA fragments upstream of the transcription initiation site of CHCHD2, N1 (between −1871 and +93), N2 (between −1691 and +93), N3 (between −257 and +93) and N4 (between −157 and +93) were inserted into the Kpn I and Bgl II restriction sites of a pGL4.10 Basic vector (Promega Corp.). The PCR primers used were as follows: N1 forward, 5′-GG TACCCTTTGGGGGGAACAGGTGGT-3′; N2 forward, 5′-GGTACCACCCACCTAGCACATCCC-3′; N3 forward, 5′-GGTACCGTTGACCGCGAAGGACGAG-3′ and N4 forward, 5′-GGTACCTGGTTGGTTGCGCGTTGAG-3′; as well as a common reverse, 5′-AGATCTCGGCCTCCC TCTGCGTCAT-3′.

The coding sequence of CREB was amplified from the HepG2 cDNA by qPCR and was subcloned into pcDNA3.1 (−). The primers used were as follows: Forward, 5′-GAATTCCGGAGGTGTAGTTTGACG-3′ and reverse, 5′-GGATCCTTAATCTGATTTGTGGCAGT-3′.

### Transient transfection and luciferase reporter assays

The HepG2 cells were cotransfected with 0.4 *μ*g plasmid-constructed CHCHD2 promoter and 13 ng internal control plasmid phRL-TK using Lipofectamine 2000 (Invitrogen Life Technologies) according to the manufacturer’s instructions. At 24 h post-transfection, the cells were harvested and lysed in 50 *μ*l passive lysis buffer (Thermo Fisher Scientific). A fraction of the protein was subjected to a Dual-luciferase Reporter Assay System kit (Promega Corp.). The firefly luciferase activity and Renilla luciferase activity were measured sequentially using a Veritas Microplate Luminometer (Turner BioSystems, Inc., Sunnyvale, CA, USA). All transfections were performed in triplicate and the promoter activities were expressed as the mean ± standard deviation of three independent experiments.

### Electrophoretic mobility shift assay (EMSA)

For the gel shift assay, double-stranded DNA oligonucleotides were synthesized with a biotin label at the 3′-end (Invitrogen, Shanghai, China). The nuclear extracts were prepared using a Nuclear Extraction kit (Pierce Biotechnology, Inc., Rockford, IL, USA) and the protein content was measured using a BCA Protein Assay kit (Pierce Biotechnology, Inc.) according to the manufacturer’s instructions. EMSAs were performed using a LightShift chemiluminescence EMSA kit (Pierce Biotechnology, Inc.). The oligonucleotide selected for EMSA contained sequences matching a consensus CREB binding site. CREB wild-type oligonucleotide probe, 5′-GGAAGAGCAGGACGTCACGGG GACGCCTCGTCC-3′ and mutant oligonucleotide probe, 5′-GGAAGAGCAGGACCGGGGACGCCTCGTCC-3′. The nuclear extracts, containing 5 *μ*g protein, were incubated with the aforementioned oligonucleotide probes for 20 min at 25°C. For the super shift assay, 5 *μ*g anti-CREB (Abcam, Cambridge, MA, USA) was pre-incubated with the nuclear extracts for 30 min and the labeled probes were then added to the reaction. Subsequently, the DNA-protein complexes were separated using a 6.5% non-denaturing polyacrylamide gel (Life Technologies, Grand Island, NY, USA).

### Chromatin immunoprecipitation (ChIP)

A total of 1×10^6^ HepG2 cells were used for each ChIP assay. The chromatin isolation and ChIP assays were performed using an EZ-Zyme Chromatin prep kit and an EZ-ChIP kit (Millipore). The chromatin solution was immunoprecipitated with either 5 *μ*g anti-CREB (Abcam) or 5 *μ*g normal anti-immunoglobulin (Ig)G antibody and 20 *μ*l protein A agarose beads (Cell Signaling Technology, Inc., Danvers, MA, USA) overnight at 4°C. Following sequential washes, once with each of the following buffers (Buffer A, low salt wash buffer; Buffer B, high salt wash buffer; Buffer C, LiCl wash buffer and Buffer D, Tris-HCl EDTA buffer), the antibody-protein-DNA complex was eluted from the beads. Following reverse cross-link incubation, which comprised the addition of 20 *μ*l 5 M NaCl per tube (NaCl final concentration, 0.2 M). The tubes were agitated continuously and incubated at 65°C overnight to induce cross-linking. Subsequently, the protein and RNA were removed by proteinase K and RNase and a qPCR assay was performed on the immunoprecipitated genomic DNA with primers specific for the CREB binding site upstream of the transcriptional start site. The primers used were as follows: Forward, 5′-AGGACCGGAGGACAAGGTTC-3′ and reverse, 5′-CTTCCGTTCTCCGTCGTCTC-3′.

### Site-directed mutagenesis

N3 was used to perform site-directed mutagenesis of the putative CREB binding sites following the quick change site-directed mutagenesis protocol (Stratagene, La Jolla, CA, USA). The oligonucleotide incorporating mutant bases was as follows: CREB, 5′-GGACGAGGCGTCCCCGGTCCTGCTCTTCC-3′. The qPCR reaction was performed for 30 cycles (95°C for 30 sec, 55°C for 30 sec and 68°C for 10 min) following an initial denaturation at 95°C for 30 sec. The mixture of input and amplified DNA was digested directly with Dpn I and then transfected into the HepG2 cells. The nucleotide sequences (5′-GCGTGATCCCTGGTACCAGAGCTCCGCCTC-3′) of the mutant were confirmed by sequencing (Beijing AuGCT DNA-SYN Biotechnology Co., Ltd., Beijing, China).

### Statistical analysis

All of the experiments were performed at least three times. The results are expressed as the mean ± standard deviation. Statistical comparisons were made using an unpaired two-tailed Student’s t-test. P<0.05 was considered to indicate a statistically significant difference. All analyses were performed using SAS statisical software version 9.1 (SAS, Cary, NC, USA).

## Results

### Patients and clinical characteristics

Table I shows the characteristics of the 110 patients. Of these, the median age was 50.9±9.2 years and the majority of patients were male. As expected, hepatitis B virus (HBV) was the major etiology (n=105) observed in 95.5% of the total patients. The majority of patients (n=96, 87.3%) had evidence of cirrhosis. In total, >61 patients (55%) had not received antiviral therapy; however, the mean duration of HBV was 12.1±8.6 years. Vascular invasion was relatively infrequent. The AFP levels in the majority of patients with HCC was <20 *μ*g/l.

### Molecular diagnosis of HCC

The results of the staining for gene expression (CD34, GPC-3, GS, HSP70, Ki-67, PEG10 and CHCHD2) are shown in Table II. The number of specimens was limited; therefore, certain biomarkers were not determined in all samples as single staining was performed on one slide. As Table II shows, the CD34, GPC-3, GS, HSP70, Ki-67 and PEG10 biomarkers had a positive rate of >90% and the novel biomarker CHCHD2 was expressed in HCC. The positive ratio for the presence of CHCHD2 was 96.3%; however, no significant difference was observed compared with that of the other biomarkers (P>0.05). All the biomarkers exhibited highly positive rates for moderate- and low-grade HCC, whereas only CD34, CHCHD2 and PEG10 exhibited high rates for high-grade HCC. The expression levels of different biomarkers were associated with tumor size, differentiation and AFP level; however, no significant difference was observed between their expression levels (Table III).

### Expression of biomarkers in HCC specimens

**Figure 1:**
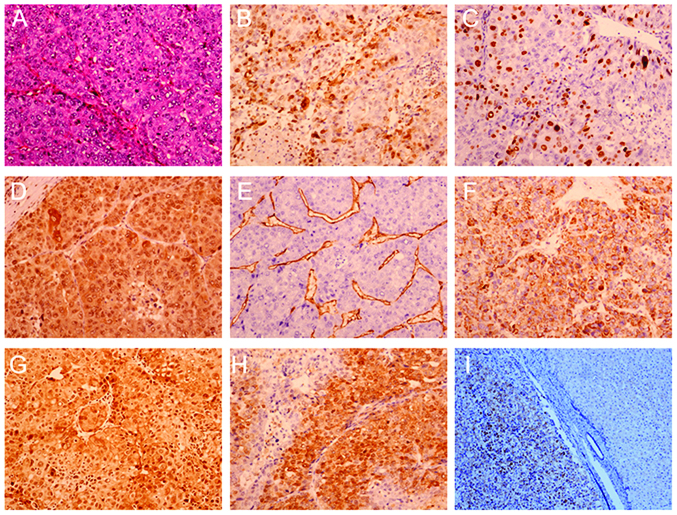
Results of immunohistochemical staining of the hepatocellular carcinoma tissues (magnification, ×40). (A) Hematoxylin and eosin; (B) HSP70; (C) Ki-67; (D) PEG10; (E) CD34; (F) CHCHD2; (G) GPC3 and (H) GS. (I) Biopsy of liver cancer stained for CHCHD2 exhibits marked, diffuse positive staining in the tumor tissue (left), but minimal staining in the adjacent normal liver tjssue (right). CHCHD2, coiled-coil-helix-coiled-coil-helix domain containing 2.

The expression of histological biomarkers was examined in the HCC specimens (Fig. 1A–H). Immunohistochemical staining for the CHCHD2 biomarker was markedly positive and diffuse throughout the tumor tissue (Fig. 1I, right) and absent from the adjacent normal liver tissue (Fig. 1I, left).

### Identification of the CHCHD2 gene

To elucidate the biological role of HVC NS2, the present study aimed to identify potential proteins which interact with NS2. SSH was introduced to establish a subtractive cDNA library of the HepG2 cells transfected with a pcDNA3.1 (−)−NS2 expression plasmid. To evaluate the efficiency of cDNA subtraction, the transcriptional expression levels of the housekeeping gene GAPDH was evaluated by RT-qPCR using the Power SYBR Green PCR Mix (Applied Biosystems Life Technologies) in the subtracted and unsubtracted cDNA libraries. The detection of GAPDH sequences for the two subtractions required 28 PCR cycles with the subtracted cDNA as the template, whereas only 18 cycles were required to amplify the GAPDH from the unsubtracted cDNA. Thus, the commonly expressed gene GAPDH was significantly depleted from the subtracted cDNA libraries. In total, 30 subtractive cDNA clones were obtained by performing a BLAST search for comparison with NCBI RefSeq, GenBank and dbEST. The CHCHD2 gene was successfully cloned from the HepG2 cell cDNA and was confirmed by sequencing. According to the NCBI database (http://www.ncbi.nlm.nih.gov/), CHCHD2 (GenBank accession no. AY605046.1) was named alternatively as coiled-c oil-helix-coiled-coil-helix domain containing 2. CHCHD2 was located at 7p11.2, containing 456 nucleotide bases and encoding 151 aas.

### Upregulation of CHCHD2 by HCV NS2

**Figure 2:**
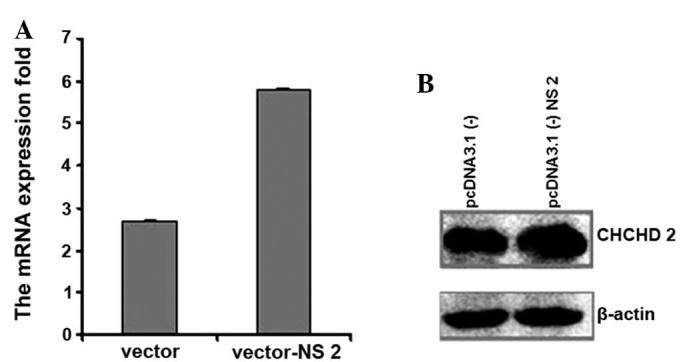
Expression of CHCHD2 was induced by the hepatitis C virus NS2. (A) Reverse transcription quantitative polymerase chain reaction analysis of the mRNA expression of CHCHD2. The mRNA expression of CHCHD2 was increased by NS2 protein in the HepG2 cells. Data is shown as the relative quantity compared with the controls following normalization with β-actin. The data represent the mean ± standard deviation of three independent experiments. (B) Protein expression of CHCHD2 by western blot analysis. The strap of CHCHD2 protein indicated that NS2 significantly upregulated the protein expression of CHCHD2. NS2, nonstructural protein 2; CHCHD2, coiled-coil-helix-coiled-coil-helix domain containing 2.

The present study demonstrated that CHCHD2 was upregulated by HCV NS2 in the SSH. In order to demonstrate whether HCV NS2 induced the expression of CHCHD2, CHCHD2 mRNA and protein were analyzed. To determine this, 4 *μ*g pcDNA3.1 (−)−NS2 was transfected into the HepG2 cells and a pcDNA3.1 (−) vector was used as a control. An increase in mRNA expression of CHCHD2 was detected (~2-fold) using RT-qPCR 24 h after the initial transfection (Fig. 2A). The whole-cell lysates were then analyzed by western blot analysis with a mouse monoclonal antibody to detect the protein levels of CHCHD2. Transfection of the cells with pcDNA3.1 (−)−NS2 caused an increase in the protein expression of CHCHD2 compared with transfection with the pcDNA3.1 (−) vector (Fig. 2B). These results suggested that NS2 upregulated the expression of CHCHD2.

### Cloning and analysis of the promoter of CHCHD2

**Figure 3:**
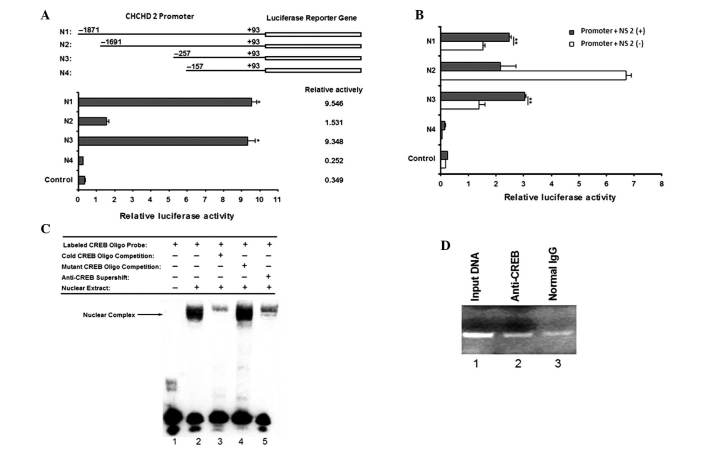
Assessment of the CHCHD2 promoter. (A) Identification of the proximal CHCHD2 gene promoter. The CHCHD2 gene promoter was constructed and pGL4.10 Basic was used as control. The ratio of luciferase activity to that of the Renilla control is shown in the graph. Values are presented as the mean ± SD of six wells; ^*^P<0.05. (B) Role of the HCV NS2 protein on the CHCHD2 gene promoter. HepG2 cells were transiently cotransfected with the reconstructed CHCHD2 promoter DNA combined with pcDNA3.1 (−)−NS2. The pGL4.10 Basic + pcDNA3.1 (−) vector was used as a control. The data represent the mean ± SD of six wells. Statistical significance was determined by an unpaired two-tailed Student's t-test; ^**^P<0.01. (C) CREB bound to the putative CREB binding site in the CHCHD2 gene promoter. With the exception of lane 1, the nuclear extracts prepared from HepG2 cells were incubated with labeled oligo-nucleotide containing the wild-type CREB binding site under various conditions. The arrow indicates the DNA/protein complex of the CREB. (D) Binding of CREB to the minimal CHCHD2 gene promoter was analyzed using a ChIP assay. Lane 1, PCR product derived from 1% of the unimmunoprecipitated genomic DNA; lane 2, PCR product derived from the DNA template immunoprecipitated by the anti-CREB antibody; lane 3, PCR product derived from the DNA template immunoprecipitated by normal IgG. A band of 159 bp containing the CREB binding site in the CHCHD2 promoter gene was amplified. The ChIP assay confirmed the binding of CREB to the CHCHD2 gene promoter DNA. CHCHD2, coiled-coil-helix-coiled-coil-helix domain containing 2; SD, standard deviation; HCV, hepatitis C virus; CREB, cyclic adenosine monophosphate response element-binding protein; ChIP, chromatin immunoprecipitation; IgG, immunoglobulin G; NS2, nonstructural protein 2.

Using the NCBI database, the present study characterized the 5′-flanking region upstream of the CHCHD2 gene. Typical TATA boxes and a high GC content was present, suggesting the possibility of a number of transcription factor binding sites within this region. To identify and analyze the promoter of CHCHD2, a 1,964 bp genomic DNA fragment was amplified from the genomic DNA, which was previously isolated from the HepG2 cells. A luciferase assay was then performed by cloning the putative promoter region of CHCHD2 into the pGL4.10 basic reporter plasmid to produce a construct termed N1, which was used as a template for further shortened CHCHD2 promoter constructs. The luciferase assays were performed 24 h after transfection of the HepG2 cells. Compared with the pGL4.10 basic plasmid, the luciferase activity of N1 increased (~27 fold), suggesting that the CHCHD2 promoter was active in the HepG2 cells. Subsequently, the transcriptional regulatory activity was determined in the promoter region. An additional three constructs containing sequentially truncated promoter fragments were generated (Fig. 3A). As shown in Fig. 3B, there was a decrease in activity when the 5′-end was shortened from N1 to N2, suggesting that the regulatory elements located between −1871 and −1691 may have acted as potential enhancers. However, there was a loss of promoter activity with N4. The serial deletion analysis therefore suggested that N3, including a 350-bp fragment of the promoter (between −257 and +93), contained the requisite sequences important for transcriptional activity.

To examine the effect of the HCV NS2 protein on the CHCHD2 promoter, transient cotransfection was performed using the pcDNA3.1 (−)−NS2 and CHCHD2 promoters in the HepG2 cells. The luciferase reporter assay results revealed that the expression of NS2 promoted the production of luciferase in N1 and N3 compared with the controls (Fig. 3B), suggesting that NS2 was responsible for the upregulated gene expression of CHCHD2 by increasing the transcriptional activity of its promoter regions between nucleotides −257 and +93.

### Specific binding of the CREB transcription factor to the CHCHD2 proximal promoter

The present study used the Promoter Scan (http://www-bimas.cit.nih.gov/molbio/proscan/) and TFSEARCH (http://www.cbrc.jp/research/db/TFSEARCH.html) databases to identify the putative transcription factor binding sites. Based on the scans for the consensus transcription factor binding motifs, one major cis-acting element, with the highest score for CREB, was present within the minimal promoter region (between −257 and +93). The CREB binding site was identified between −223 and −216. This suggested that activation of the CHCHD2 promoter by NS2 was regulated by the binding of CREB to the putative binding site.

To examine whether CREB was able to bind to the CHCHD2 promoter, EMSAs were performed. The first oligonucleotide selected for EMSA contained sequences matching a consensus CREB binding site. The nuclear extracts from the HepG2 cells exhibited marked binding to a wild-type probe containing a CREB binding site (Fig. 3C; lane 2). Competition experiments were then performed using unlabeled probes and mutant probes (Fig. 3C; lanes 3 and 4). The DNA-protein complex was specific, as it was markedly out-competed by a 100-fold excess of the unlabeled probe, whereas the mutant probe, containing a 4-bp deletion, failed to compete. However, the nuclear complex likely contained CREB as part of a larger protein complex, since the complex in lanes 3 and 5 (Fig. 3C) was reduced by the unlabeled probe and antibody, respectively. These results suggested that CREB was able to bind to the CHCHD2 minimal promoter.

To assess whether CREB was able to bind to the CHCHD2 proximal promoter *in vitro*, ChIP assays followed by qPCR were performed. The results demonstrated an increase of CREB on N3 compared with those in the controls, which were precipitated with IgG (Fig. 3D). A 159-bp band containing the CREB binding site in the CHCHD2 promoter gene was amplified following qPCR amplification. By contrast, the IgG immunoprecipitated products did not contain the CHCHD2 promoter DNA sequences (Fig. 3D; lane 3). As a positive control, the isolated genomic DNA input was directly used for qPCR analysis and the corresponding 159-bp fragment was also identified (Fig. 3D; lane 1). These data revealed that CREB can specifically bind to the CHCHD2 promoter.

### Functional analysis of the effect of CREB on CHCHD2 promoter activity

**Figure 4:**
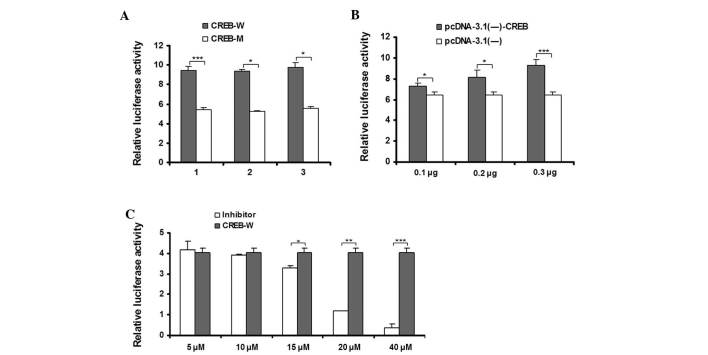
CREB-induced transcriptional activity of CHCHD2. (A) Relative luciferase activity of N3 and its mutant. (B) Expression of the exogenous CREB-induced dose-dependent increase in the relative luciferase activity of N3 with 0.1, 0.2 and 0.3 *μ*g pcDNA3.1 (−)−CREB. (C) H89 treatment resulted in a reduction in luciferase activity of N3. The HepG2 cells were incubated in Dulbecco’s modified Eagle’s medium with H89 at the indicated concentrations. Values are presented as the mean ± SD of three independent experiments. Statistical significance was determined by an unpaired two-tailed Student’s t-test. ^*^P<0.05; ^**^P<0.01; ^***^P<0.001. CREB, cyclic adenosine monophosphate response element-binding protein; CHCHD2, coiled-coil-helix-coiled-coil-helix domain containing 2; N3, DNA fragment of CHCHD3 between nucleotides-257 and +93; SD, standard deviation; W, wild-type; M, mutant.

To confirm whether the transcription factor CREB has a functional role in the activation of the CHCHD2 promoter, site-directed mutagenesis of the CREB binding site within N3 was performed and the luciferase activity between the wild-type and CREB mutant binding site were compared using transient transfection experiments. As shown in Fig. 4A, The mutations of the CREB site had a marked effect on the promoter activity, as demonstrated by a 40%-decrease in luciferase activity. The mutational analyses indicated that abrogation of the CREB binding site was sufficient to disrupt the activity of the CHCHD2 promoter.

To assess the effect of CREB on the expression of the CHCHD2 promoter, co-transfection experiments of N3 with an expression plasmid for CREB were performed. As shown in Fig. 4B, the transcriptional activity of the CHCHD2 promoter was not altered in the control group. By contrast, the transcriptional activity was markedly induced by pcDNA3.1 (−)−CREB. The presence of CREB (between 0.1 and 0.3 *μ*g) induced a dose-dependent increase in luciferase activity, suggesting the importance of CREB in regulating the gene expression of CHCHD2.

With respect to PKA, the phosphorylation of CREB was shown to increase the transcription of cAMP-responsive genes and the PKA inhibitor H89 has been used to inhibit CREB phosphorylation (21). Therefore, H89 was used to evaluate the effect of CREB on the transcriptional activity of CHCHD2. The inhibitor concentrations were based on previous studies, which established effective levels, all involving HepG2 cells (22–24). As shown in Fig. 4C, the N3 luciferase activity was disrupted in the HepG2 cells in the presence of H89. These results suggested that CREB is important for maintaining the transcriptional activity of CHCHD2.

## Discussion

HCV NS2 is a 217 aa-long cysteine protease, which cleaves the NS2–3 junction in cooperation with the N-terminal 180 aas of NS3, forming serine-type protease, likely in a rapid intramolecular reaction (5,25). NS2 potentially modulates the host cell environment during HCV infection through interference with gene expression and through the induction of hepatocellular apoptosis, particularly as CHCHD2 localizes to the mitochondria (26). A potential role for NS2 in the modulation of the host cell environment has potentially important implications for the establishment of persistent infection and the pathogenesis of chronic HCV infection (8,27). Several HCV proteins have been implicated in the modulation of cell signaling and apoptosis, including core, E2, NS5A and NS2. The expression of these proteins in the liver prevents the release of cytochrome c from the mitochondria and suppresses the activity of caspase 9 and caspase 3/7 without affecting caspase 8 (28). Therefore, the HCV proteins may be involved in the mitochondrial intrinsic apoptotic pathway, which involves increased mitochondrial membrane permeabilization and the release of pro-apoptotic factors, resulting in cell death (29). Numerous interactions between NS2 and viral or cellular proteins have been reported. To further examine the function of NS2, the present study used SSH to identify CHCHD2. At present, no correlation has been observed between CHCHD2 and NS2. The inhibition of NS2-induced apoptosis may, in part, be due to its ability to induce the expression of CHCHD2, although, to the best of our knowledge, no previous study has focused on this possible pathogenic mechanism.

CHCHD2 is connected to 83 genes in the co-expression network, which are involved in glycolysis and translation (30). Together, the findings of the present study suggested that CHC H D2 is important in translation in human cells. Previously, the gene expression of human CHCHD2 has been determined by transcriptome analysis to be increased in certain types of cancer tissue compared with that in normal tissues, suggesting that CHCHD2 may be associated with the progression of cancer (31,32). On the basis of a previous study, CHCHD2 is important in enhancing cell migration-promoting activity (13), instead of promoting cell proliferation. Mitochondrial oxidative phosphorylation (OxPhos) is central to energy homeostasis and human health by serving as the primary generator of ATP in the cell (20,33–36) and knockdown of CHCHD2 resulted in OxPhos deficits.

In the present study, to investigate the function of CHCHD2, the mechanism of CHCHD2 transcriptional regulation was examined. The NS2 transregulation gene was identified using SSH and, according to RT-qPCR and western blot analyses, NS2 was able to upregulate the expression of CHCHD2. In the present study, a region of the CHCHD2 promoter, which was 1,964 bp upstream of the transcription start site, was cloned, the promoters were deleted, the plasmid which was reconstructed, was constructed in a stepwise fashion and analyzed for luciferase activity in the HepG2 cells. A minimal promoter sequence, spanning between nucleotides −257 and +93, was sufficient to drive the expression of CHCHD2.

The present study identified a novel gene coding for the NS2 transregulated protein CHCHD2. Previous studies have demonstrated that CHCHD2 is a member of a protein family that contains the (coiled coil 1)-(helix 1)-(coiled-coil 2)-(helix 2) (CHCH) domain (23); however, no previous studies have reported an interaction between CHCHD2 and HCC.

To date, immunohistochemical analysis of the expression of CHCHD2 has not been performed in large scale studies of human tumors. The present study identified the overexpression of CHCHD2 in liver tissues, which was particularly evident in early and well-differentiated HCC compared with the surrounding non-cancerous liver tissue. Therefore, these results suggested that CHCHD2 may be a novel and useful diagnostic biomarker for early HCC.

## Figures and Tables

**Table I:** Tumor and patient characteristics.

A, Patient characteristic	Number (%)
Age (years)	
Mean	50.9±9.2
Range	30–80
30–40	15 (13.6)
41–50	39 (35.4)
51–60	43 (39.1)
>60	13 (11.8)
Gender	
Male	87 (79.1)
Female	23 (20.9)
Viral etiology	
Hepatitis B	105 (95.5)
Hepatitis C	5 (4.5)
Virus found time	
Mean (years)	12.1±8.6
Longest	40 years
Shortest	1 day
Antiviral time (years)	
≤1	16 (14.5)
1–3	10 (9.1)
>3	23 (20.9)
No antivirus	61 (55.5)
Outcome (3 years)	
Alive	77 (70.0)
Deceased	7 (6.4)
Unknown	26 (23.6)
HBV DNA level (cp/ml)	
<500	51 (46.4)
≥500	54 (49.1)

**B, Tumor characteristics**	

Diameter of tumor (cm)	
<3	48 (43.6)
3–5	45 (40.9)
>5	17 (15.5)
Cirrhosis	
Yes	96 (87.3)
No	14 (12.7)
AFP(ng/ml)	
≤20	47 (42.7)
20<AFP≤200	26 (23.6)
200<AFP≤400	20 (18.2)
>400	17 (15.5)
Envelope	
Complete	49 (44.5)
No envelope	27 (24.5)
Unknown	34 (30.9)
Lesion position	
Right lobe	83 (75.5)
Tumor characteristic	Number (%)
Left lobe	26 (23.6)
Caudate lobe	1 (0.9)
Vascular invasion	
Positive	46 (41.8)
Negative	64 (58.2)
Differentiation of tumors	
High grade	11 (10.0)
Moderate grade	63 (57.3)
Low grade	36 (32.7)
Hepatitis B	105
HBeAg(+)	46 (41.8)
HBeAg(-)	59 (53.6)

Tumor size was measured based on length of largest tumor nodule. HBV, hepatitits B virus; AFP, α-fetoprotein; HBeAG, hepatitis B e antigen.

**Table II:** Expression of gene markers associated with differentiation.

Gene marker	Positive, n (%)				Negative, n (%)			
	Total	Differentiation			Total	Differentiation		
		High	Moderate	Low		High	Moderate	Low
CD34	77 (100)	6 (100)	45 (100)	26 (100)	0 (0.0)	0 (0.0)	0 (0.0)	0 (0.0)
GPC-3	75 (94.9)	5 (83.3)	45 (95.7)	25 (96.2)	4 (5.1)	1 (16.7)	2 (4.3)	1 (3.8)
GS	37 (94.9)	4 (80.0)	19 (100)	14 (93.3)	2 (5.1)	1 (20.0)	0 (0.0)	1 (6.7)
HSP70	38 (97.4)	4 (80.0)	19 (100)	15 (100)	1 (2.6)	1 (20.0)	0 (0.0)	0 (0.0)
Ki-67	76 (96.2)	5 (83.3)	46 (97.8)	25 (96.2)	3 (3.8)	1 (16.7)	1 (2.1)	1 (3.8)
CHCHD2	105 (96.3)	10 (90.9)	60 (95.2)	35 (100)	4 (3.7)	1 (9.1)	3 (4.8)	0 (0.0)
PEG10	109 (99.1)	11 (100)	62 (98.4)	36 (100)	1 (0.9)	0 (0.0)	1 (1.6)	0 (0.0)

**Table III:** Expression of different gene markers associated with tumor size, differentiation and AFP level.

	Size (cm)	Differentiation						AFP					
		High		Moderate		Low		<200		200–400		>400	
	
		+	–	+	–	+	–	+	–	+	–	+	–
CD34	<3	4	0	23	0	10	0	25	0	5	0	7	0
	3–5	2	0	14	0	11	0	20	0	4	0	3	0
	>5	0	0	8	0	5	0	6	0	2	0	5	0
GPC-3	<3	4	0	23	1	91	1	24	2	5	0	7	0
	3–5	1	1	13	1	11	0	18	2	5	0	2	0
	>5	0	0	9	0	5	0	6	0	3	0	5	0
GS	<3	3	0	10	0	5	0	13	0	0	0	5	0
	3–5	1	1	5	0	7	1	8	2	3	0	2	0
	>5	0	0	4	0	2	0	1	0	0	0	5	0
HSP70	<3	3	0	10	0	5	0	13	0	0	0	5	0
	3–5	1	1	5	0	8	0	9	1	3	0	1	0
	>5	0	0	4	0	2	0	1	0	0	0	5	0
Ki-67	<3	4	0	23	1	10	0	25	1	5	0	7	0
	3–5	1	1	14	0	11	0	19	1	5	0	2	0
	>5	0	0	9	0	1	1	5	1	3	0	5	0
CHCHD2	<3	5	0	32	0	1	0	34	0	7	0	7	0
	3–5	5	1	17	3	18	0	27	4	10	0	4	0
	>5	0	0	11	0	6	0	8	0	3	0	6	0
PEG10	<3	5	0	32	0	11	0	34	0	7	0	30	1
	3–5	6	0	19	1	19	0	30	1	10	0	4	0
	>5	0	0	11	0	6	0	8	0	3	0	6	0

AFP, α-fetoprotein.
